# Teratogenic Effects of Long Term Consumption of Potassium Benzoate on Eye Development in Balb/c Fetal Mice

**Published:** 2013-04

**Authors:** Mohammad Afshar, Seyed Adel Moallem, Jina Khayatzadeh, Marziyeh Shahsavan

**Affiliations:** 1 Department of Anatomy, Faculty of Medicine, Birjand University of Medical Sciences, Birjand, Iran; 2 Medical Toxicology Research Centre, Mashhad University of Medical Sciences, Mashhad, Iran; 3 Department of Pharmacodynamics and Toxicology, school of Pharmacy, Mashhad University of Medical Sciences, Mashhad, Iran; 4 Pharmaceutical Research Centre, Mashhad University of Medical Sciences, Mashhad, Iran; 5 Department of Biology, Faculty of Basic Sciences, Mashhad Branch, Islamic Azad University, Mashhad, Iran

**Keywords:** Eye Development Mice Fetuses, Potassium Benzoate, Teratogenicity

## Abstract

***Objective(s):*** Potassium benzoate (PB) is used as a substitute preservative for sodium benzoate primarily in dietetic foods where the sodium content is minimized. There are few reports about the teratogenic effects of PB. The purpose of this study is to investigate the teratogenic effects of PB on eye development in balb/c mice fetuses.

***Materials and Methods:*** Thirty female mice were divided to two experimental and one control groups (N=10/grp). Experimental groups I and II received daily intraperitoneal (IP) injections of 280 and 560 mg/kg of PB, respectively; while the control group received normal saline. Injections were done for 10 days before mating and from gestation days (GD) 6 through 15. Dams were Caesarean sectioned on GD 18. Eye development was externally examined. After measuring weight and crown-rump length of the fetuses, the tissue sections of the eyes were prepared and stained with hematoxylin-eosin. Histopathologic and morphologic changes were recorded. The data was analyzed by ANOVA and Mann Whitney statistical tests using SPSS (version 15).

***Results: ***Fetuses with eye malformations observed in both experimental groups of I and II. The incidence of these malformations was significantly increased in fetuses of experimental group II as compared with the control group (*P<*0.05). Histopathological evaluations of the malformed eyes showed deformed lens, retinal folds with undeveloped layers associated with hemorrhage.

***Conclusion:*** Our results suggest that PB can induce teratogenic effects on the eye development of mice fetuses. Therefore, more concise studies are needed regarding its specific and general effects conducted.

## Introduction

Benzyl alcohol, benzoic acid and its sodium and potassium salts can be considered under one category regarding human health, as they are all rapidly metabolised and excreted via a common pathway within 24 hr. The systemic toxic effects of similar nature (liver, lung, and kidney) have been observed (1). The study of Ping Yu *et al* (2009) has shown that a semisynthetic 2-benzoylaminobenzoic acid derivative, methyl 2-(2-fluorobenzamido) benzoate (DSM-RX78), attenuates hemorrhagic shock-induced lung injury in rats (2). 

Sodium benzoate (SB) and potassium benzoate, in low concentrations, are used in aqueous solutions. PB serves as a substitute for SB, mostly in dietetic meals where the sodium content needs to be minimized (3, 4). Unfortunately, little information is available about potassium benzoate (PB)-induced toxicity and teratogenicity during early fetal development. However, some information has been reported from studies investigating other benzoate salts. To evaluate the teratogenic effects induced by physical and chemical agents, various test systems have been described in bacteria, in mammalian cells, and in plants; for example, the effects of SB in chronic exposure with mammals were limited to reduced food intake and growth (5,6). Vernole *et al* (1987) have studied the cytogenetic effects of 1-p-(3-methyltriazeno) benzoic acid potassium salts in human lymphocytes. A dose-dependent increase of chromosome breaks in human lymphocytes after exposure to 1-p-(3-methyltriazeno) benzoic acid potassium salts has also been reported (7,8). 

It has also been reported that sodium benzoate at concentrations below 100 pg/ml and exceeding 500 pg/ml inhibits intracellular protein and DNA synthesis (9). 

The study of Mpountoukas *et al* (2008) has shown that benzoic acid enhances chromosomal aberrations, sister chromatid exchanges and micronucleus prevalence (200 and 500 lg/ml) in human lymphocytes without manipulating the pH of the medium (10). The mechanism of benzoic acid-mediated mutations in human lymphocytes has not been ascertained yet. However, genotoxicity might be affected by the inhibition of the actuation of XRCC1, PARP-1 and DNA LIG3 proteins responsible for DNA repair or the inhibition of OP18 stathmin activity which adjusts microtubules (11).

Benzoic acid, its sodium and potassium salt and salbenzyl alcohol are irritating to the eye (1), but, there is not any information about the teratogenic effects of these preservatives on eye development. On the other hand, some major malformations of human eye, such as microphtalmia and anophtalmia, are examples of phenotypes that show no Mendelian inheritance pattern. There is some evidence that mutation in homeobox gene, OTX2, may be involved in inducing these phenotypes. Bilateral anophthalmia to some retinal effects and pigmentary retinopathy are also related to the expression pattern of OTX2 gene in human embryo (12).Regarding the consumption of PB, as it can have mutagenic activity, we conduct the current study to specifically assess the potential teratogenic effects of the food preservative, PB, on the embryonic eye development in the pregnant mice.

## Materials and Methods

*Animals treatments*

Virgin female Balb/c mice, weighing 28-30 g (8-9 week old) were used in this study. The animals were maintained in a climate-controlled room under a 12 h alternating light/dark cycle (9.00- 21.00 hr light), 20.1 to 21.2 ^0^C temperature and 50 to 55.5 % relative humidity. Dry food pellets and water were provided ad libitum. After two weeks of acclimation to the diet and the environment, three females were caged with a male of the same strain overnight. The presence of a vaginal plug in the following morning confirmed that mating had occurred and was designated as Gestation Day (GD) 0. Maternal weights were measured throughout the experiment. (10 days before mating and GD0 until GD18). Thirty pregnant mice were randomly divided into 3 experimental groups (10 mice in each group) receiving daily intraperitoneal (IP) injections of 280 mg/kg/day PB (Group I), 560 mg/kg/day PB (Group II) and normal saline (Group III Control). All injections were done for 10 days before mating and from GD 6 – 15. There are few studies that have reported the teratogenic effects of potassium benzoate. However, the teratogenic effects of SB in rats using almost the same doses as in this study have been reported (1,13). PB powder was purchased from Sobhan Darou Pharmaceutical Company in Tehran, Iran. Approval for this study was obtained from the Mashhad University of Medical Sciences Animal Care and Ethics Committee.

*Fetal assessment*

On GD 18, pregnant mice were killed using ether anesthesia and the uterus was opened and the umbilical cord cut close to the fetus; each fetus was then weighed. Fetuses were assessed as either alive or dead. Fetuses were examined externally and crown-rump length measured. Fetuses with eye abnormalities were separated from normal fetuses. 

*Histological analysis*

Specimens were processed for histology by fixation in Bouin’s solution or 10% paraformaldehyde in phosphate-buffered saline, followed by paraplast embedding and sectioning. Sections 7–10μm thick were stained with Mayer’s hematoxylin-eosin (HE) stain (14). Histopathologic sections were observed for any abnormality and were recorded.

*Statistics*

For the numbers of implantations, fetal body weight and the crown-rump length of each fetus, the units of analysis were reported as mean ± SD. Tukey test was done after ANOVA between treated groups and each treated group with the control group. The frequency of hemorrhage in the surface of the eye of each fetuse, the percentage of hemorrhage recorded between the treated groups and each treated group with control were analyzed using the Mann Whitney U test. The frequency of each category was 5 or more, with Fisher’s direct probability test for other cases. The unit of frequency analysis was fetuses. Statistical analyses were carried out by the means of SPSS (ver. 16). Differences were considered significant at *P<*0.05.

## Results

*Fetal body weight and crown-rump lenghts with fetal hemorrhage (* Table I *)*

The mean fetal body weight of both groups I (0.71 ±1.07 g) and II (0.41±0.07 g) were significantly reduced compared to the control group (1.19± 0.31 ) (*P<*0.05). The mean fetal body weight of group II was significantly decreased as compared with group I (*P<*0.05). The crown-rump lengths of fetuses were also reduced significantly in both groups I (17 ±1.6 mm) and II (15.34 ±1.4 mm) as compared with the control group (21.4±0.61 mm). The mean of the crown-rump length of group II was significantly decreased compared with the experimental group I (*P<*0.05). 

Fetuses with eye hemorrhage

**Figure 1:**
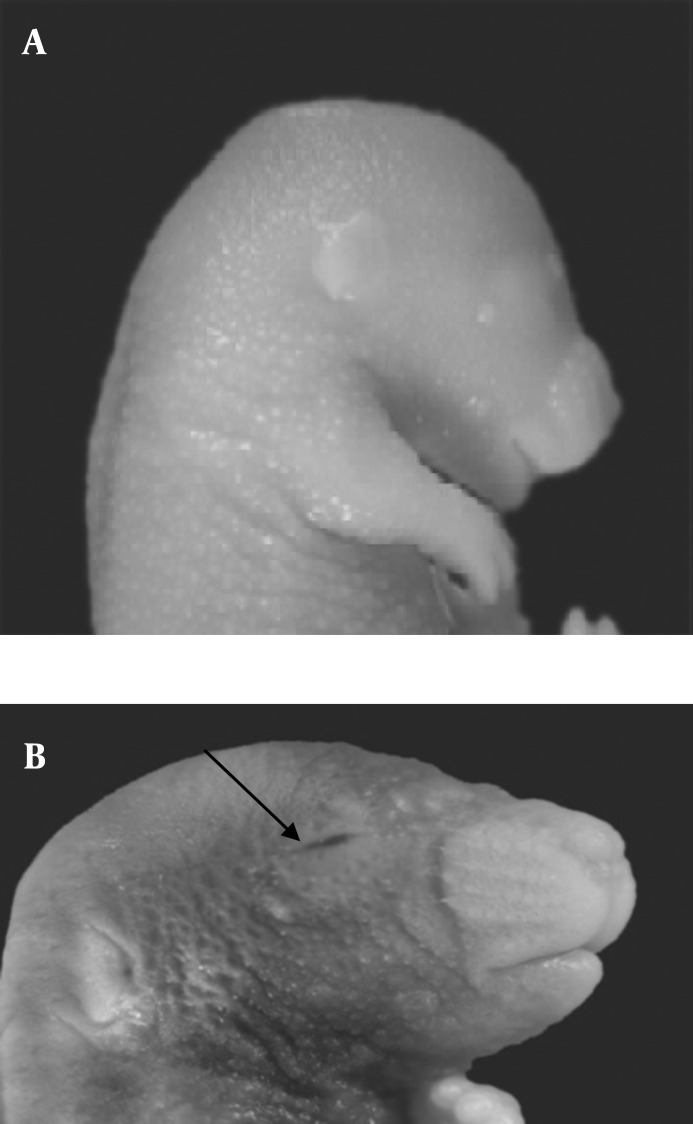
Normal mouse fetus in control group; B) Mouse fetus with eye hemorrhage in experimental group II (with arrow) viewed under stereo research microscope (10x)

Morphological study showed eye malformation, in the form of hemorrhage in the palpebral fissure of some fetuses in the both experimental groups I and II (Figure 1). The incidence of this malformation(6.8%) significantly increased in the fetuses of experimental group II as compared with fetuses in experimental group I (1.1%) and control group (*P<*0.05) .

Histopathological findings

**Figure 2:**
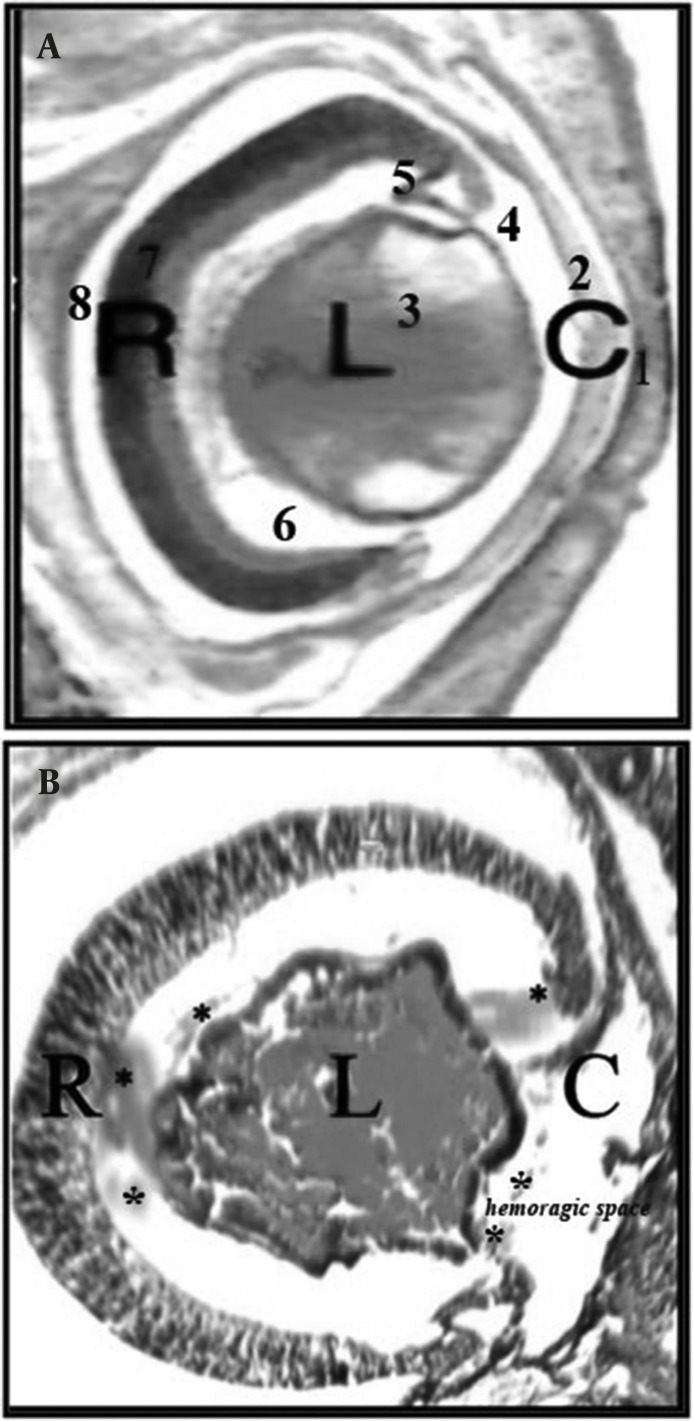
A) Tissue section from normal embryonic eye in control group; B) Tissue section from embryonic eye with hemorrhage in experimental group II detected with deformed lens, retinal folds with undeveloped layers associated with hemorrhage( with*) and corneal folds with absence of surface epithelium. (C = cornea, L= lens, R= retinal) (100x H&E). 1. Fused eyelids, hyaloid artery; 2. Cornea; 3. Lens; 4. Anterior chamber; 5.Primitive iris; 6. Vitreous humor; 7.Retinal; 8. Intraretinal space

Our histopathological finding on the embryonic eye of fetuses in the two experimental groups ( I and II )showed deformed lens in a way that the polarity of lens fibers could not be distinguished; there were also unidentified inner and outer nuclear layers of the retina and some ruptures in the undeveloped corneal layers and iris. Some hemorrhagic features were also observed in the near hyaloid artery, primitive iris and in front of corneal folds (Figure 2). 

**Table 1:** Cesarean Section Parameters on GD 18 in Balb/c Mice Treated With Potassium benzoate

Parameters	Control		Treatment group PB(mg/kg)	
		NS	280	560
Litters	No.	10	10	10
Implants	No.	109	100	118
Resorbed Fetuses	No. (%)	0(0)	**/*(1010(	**/* (27.26)31
Live fetuses	No. (%)	109(100)	(90)90	87(73.72)
Fetuses with eye malformation	No. (%)	0(0)	1(1.11)	6(6.89)**/*
Fetal body weight(g)	Mean ±S.D	0.31 ± 1.19	*/**1.07 ± 0.71	*/**0.07 ± 0.41
Crown-rump length of live fetuses(mm)	Mean ±S.D	0.61± 21.4	17±1.6**/*	1.4**/*±15.34

Litter = the number of litters having fetuses with finding (the percentage of total litters);

Fetus = the number of fetuses with finding (the percentage of total fetuses)

*P<0.05 vs. control

**P<0.05 vs. Experimental groups.

## Discussion 

In the present study, the potential teratogenic effects of PB on embryonic eye development were examined. The results demonstrated the occurrence of the extensive hemorrhage of the embryonic eye, deformed lens and retinal folds with undeveloped layers as a result of the exposure of pregnant mice to PB. 

Because of the complexity of eye development, many congenital anomalies occur, but most of them are not common. Several environmental teratogens can induce congenital eye defects. Some investigators have shown clear evidence of an association between the consumption of some antiepileptic drugs during pregnancy and congenital eye malformation such as, anophthalmia, microphthalmia, or coloboma of the iris or optic disk (15, 16). Our previous study showed that intraperitoneal administration of carbamazepine at clinically comparable doses during organogenesis can induce eye malformations in mice. It is noteworthy that CBZ is metabolized through the arene oxide pathway by which xenobiotics can be hydroxylated through epoxide intermediates (15, 17). By covalent binding to macromolecules, epoxides may have mutagenic or teratogenic properties (15). Additionally, the antiepileptic drug Valproic acid (VA) has been implicated as a human teratogen in both retrospective and prospective studies (16). VA also can induce open eye defects in some of the mice fetuses. One of the probable mechanisms that has been suggested by many researchers is the interference of this drug with folate metabolism. Localized folate deficiencies in rapidly dividing tissues can result in ineffective DNA synthesis altering cell metabolism and replication. On this basis, VA can disrupt the cell cycle and that cellular proliferation is essential for normal eye development (18,16). Furthermore, it has been reported that the PAX6 gene is a paradigm for our understanding of the molecular genetics of mammalian eye development (19). Defects in the PAX6 gene expression in mice have aniridia-like iris anomalies (20), corneal opacities and lens-corneal adhesions that resemble Peters’ anomaly (21,22). Detailed histologic studies of neonatal mice with Peters-like defects revealed that the lens frequently fails to separate from the cornea (23). Moreover the study showed that OTX2 loss-of-function mutations are associated with a broad spectrum of ocular phenotypes, ranging from bilateral anophthalmia to mild microphthalmia with retinal abnormalities (12). Vernole *et al* (1987) have studied the cytogenetic effects of 1-p-(3-methyltriazeno) benzoic acid potassium salts in human lymphocytes. A dose-dependent increase of chromosome breaks in human lymphocytes after exposure to 1-p-(3-methyltriazeno) benzoic acid potassium salts has also been reported (7,8). 

Consequently, PB and decreased cell proliferation and PAX6, OTX2mutations delayed normal eye development.

 We also found an evidence of hemorrhagic features in some locations of eye developing structure. It was reported in a study that SB-treated zebra fish, larvae exhibited phenotypic malformations, which might have resulted from diminished diamine oxidase (DAO) enzymatic activities (24). DAO transcripts were identified in neuron cells, gut, pronephric tubes and the anterior segments of pronephric ducts in zebra fish (25). In another study, SB caused diarrhea and hemorrhage in the intestines of rats (26), implying that gut lesions might be the most prevailing complications caused by SB exposure in adult zebra fish(24). Similar SB-induced intestinal hemorrhage was also observed in rats following exposure to PB (24). Based on these observations, it is proposed that the results depicted in the present study could replicate SB toxicity. 

 This may have originated from hemorrhagic shock (HS) following the start of an inflammatory cascade that is essential for the survival after successful resuscitation. There exists an intricate sequence of interrelated molecular events including shock-dependent and reperfusion-dependent reactions that propose a novel approach for the adverse effects of HS. Shock-dependent starting mechanisms comprise the induction of inducible nitric oxide synthase (iNOS), cyclooxygenase (COX)-2, and CD14, acting as a catalyst for subsequent post-resuscitation phenotypic variations. The early immediate reactive genes for iNOS and COX-2 intensify the inflammatory response via rapid and excessive generation of nitric oxide (NO) and prostaglandins. The transcription agent, hypoxia-inducible factor-1 (HIF-1), might adjust the induction of iNOS during the ischemic phase of shock. NO is one of the leading signaling molecules involved in redox-sensitive mechanisms, such as the downstream activation of nuclear factor (NF)-κB. NO-dependent NF-κB activation promotes the induction of inflammatory cytokine expression during the reperfusion phase. Peroxynitrite-mediated direct toxicity and NO-mediated inflammatory toxicity give rise to injury of organs (27,28). We hypothesize that, upon analogous mechanisms, PB leads to the activation of the inflammatory pathway, following organ injury and hemorrhage. 

Mice exposed to ethylnitrosourea (MNU) midgestation resulted in damages to proliferating cells via macromolecule alkylation and production of reactive oxygen species (ROS) (29). Elevated ROS in mice alleviated the intensity of retinal complications and prevented, by unknown mechanisms, fetal Pax-3 gene expression that is vital in the blockage of neural tube development (30). Increased ROS down-regulates bcl-2 (anti-apoptotic) gene expression in proliferating embryonic tissues in rats (31). Some benzoate derivatives like SB appear as free radical scavengers in human (32);whereas other mechanisms possibly causing embryonic hemorrhage and eye tissue disorders following PB exposure, might result from the induction of potentially detrimental ROS levels in embryonic tissues (such as eye) and inhibit embryonic gene expression like Pax-3 or alternative genes needed for blood clotting. The results of our study are similar to the hyperkalemia which has been reported to cause intra-ventricular hemorrhage in preterm infants (33). Researchers have conjectured that low systemic blood flow could be a significant factor in the pathogenesis of hyperkalemia in preterm infants through declined urinary output and reduced K+ secretion (34). It is also believed that hemolytic activity of the confined intra-ventricular blood clot may contribute to a K+ load surplus in the intra-ventricular space (33).

 In our study, we have found that mouse fetuses exposed to PB exhibit markedly decreased weight and crown-rump lengths. However, no effects have been seen in adult mice exposed to 280 and 560 mg/kg/day of PB for 20 days (data not displayed). Consequently, it is hypothesized that mouse embryos (or fetuses) are more sensitive to PB compared to adults. The effects of chronic exposure for 35 days to benzoic acid and SB in Swiss albino mice are limited to reduced food intake and declined growth (26). Declined fetal body weight, increased utero deaths, and general fetal abnormalities such as dilated renal pelvis, skeletal variations and retarded ossification, have been observed in pregnant rats injected by IP with 100–1000 mg/kg of SB (35). 

## Conclusion

According to these findings, PB additives cause histophatologic effects in eye of mice fetuses; however, the mechanisms of this activity are unknown and require further investigation. 
